# Histochemical and immunohistochemical study of mucinous rectal carcinoma

**Published:** 2017

**Authors:** CV Lungulescu, S Răileanu, G Afrem, BS Ungureanu, DN Florescu, IA Gheonea, S Șovăilă, Ș Crăițoiu

**Affiliations:** * Oncology Clinic, County Clinical Emergency Hospital of Craiova, Romania; ** Department of Medical Expertise and Recovery of Work Capacity, Dolj, Romania; *** Research Center of Gastroenterology and Hepatology, Craiova University of Medicine and Pharmacy, Craiova, Romania; **** Centre Hospitalier de Sedan, France; Internist.ro Clinic, Brasov, Romania; ***** Department of Histology, Craiova University of Medicine and Pharmacy, Craiova, Romania

**Keywords:** colorectal cancer, histopathological and immunohistochemical profile, signet ring, mucinous rectal carcinoma, mucins

## Abstract

Colorectal cancer (CRC) is a major health problem worldwide. The objective of our study was to assess the histopathological (HP) and immunohistochemical (IHC) profile of mucins from signet ring (SR) and mucinous rectal carcinoma, while evaluating their value as a prognostic factor and muco-secretive ability. The HP study (76 cases) included 4 categories of patients: pure mucinous (PM), mixed mucinous components (MM) (50-80% of the tumor cells), mixed mucinous components (Mm) (< 50% of the tumor cells) and signet ring (SR). The IHC study consisted of a total of 30 cases of MRC and was processed by the ABC/ HRP technique. The antibodies used have addressed their muco-secretive capacity: MUC1, 2 and MUC5AC. MRC cases were more frequent in the sixth decade, with a median age of 57.3 years. It could be noted that MRC tended to develop at younger ages. For the MP variant, the gender ratio was 1.37 in favor of men, while for the MM variant it was 1.16, 1.31 for the Mm and 1.6 in the case of signet ring type. Most of the MRC were moderately differentiated forms, except for the SR form, poorly differentiated forms predominating. Well-differentiated forms were the most underrepresented, being more common in the Mm version. Regarding the biochemical type of mucin, MP and SR were characterized by acid mucins and sialomucin, while in the Mm type, there was a balance of acidic and neutral mucins. The prevalence of mucin acids, respectively sulfomucin, was characteristic to younger ages and poor prognosis.

## Introduction

Colorectal cancer (CRC) is a major health problem worldwide, being the fourth cancer in men and the third in women. In recent decades, CRC has shown elevated incidence rates especially in developed countries, where obesity is associated with an increased consumption of high calorie foods and reduced physical activity [**1**].

Moreover, the death rate is on a decreasing trend. This may be explained by treatment improvements, which have increased the survival rate and also by the early detection of the disease, as a result of the screening programs [**2**].

Mucinous rectal carcinomas represent a small amount of all CRC and over 50% of all the colorectal mucinous carcinomas [**3**,**4**]. Compared to the other varieties of CRC, the mucinous version and the “signet ring” cell version have a worse prognosis, being diagnosed in more advanced stages of the disease, with a much higher rate of lymphatic metastasis, serous infiltration, and peritoneal dissemination. In addition, these two types of carcinomas have a higher rate of local extension, which leads to a lower chance for curative resection and decreasing the overall survival rate [**5**]. 

According to the World Health Organization, mucinous carcinomas require more than 50% of extracellular mucin so that the tumor may be documented [**6**]. Mucin secreting tumors have been noted in different organs such as pancreas, prostate, breast, this specific profile providing certain characteristics that allow the histopathological differentiation from the more frequent cancer types [**7**-**10**]. 

Our objective was to assess the histopathological and immunopathologic profile of mucins from the signet ring and the rectal mucinous carcinoma while evaluating their value as a prognostic factor and muco-secretive ability.

## Material and methods

The histopathological material, 76 cases selected from 2012 to 2013, came from the Pathology Department of the County Clinical Emergency Hospital of Craiova and was represented by archived paraffin blocks. We performed hematoxylin-eosin, Masson trichrome, PAS Alcian-Blue, Blue Alcian-colloidal iron, argentic impregnation staining. The histopathological study followed the main microscopic morphological characteristics of rectal mucinous carcinomas. The histopathological diagnosis was performed in accordance with the WHO criteria for the diagnosis of colorectal tumors (2000). 

Rectal mucinous adenocarcinoma was divided into 4 categories: pure mucinous (PM), mixed mucinous components (MM) (50-80% of the tumor c ells), mixed mucinous components (Mm) (< 50% of the tumor cells) and signet ring (SR). These subdivisions were made by a morphometric assessment of the surface occupied by mucin/ signet ring cell on the cross section of the resection piece stained with HE, with the help of NISE elements software (Nikon). 

The immunohistochemical study consisted of a total of 30 cases of rectal adenocarcinoma with mucinous component and was processed by the ABC/ HRP (avidin complexed with biotinylated peroxidase) technique. The antibodies used in this study have addressed their muco-secretive capacity: MUC1, 2 and MUC5AC.

## Results

In the investigated cases, the highest incidence was for Mm, followed by MP and MM forms, and, on the last place, the SR version.

Mucinous rectal carcinoma cases were more frequent in the sixth decade of life, with a median age of 57.3 years. In comparison with conventional rectal adenocarcinoma whose mean age of development is 63.2 years, it can be noted that the mucinous types tend to develop at younger ages, at least nine years earlier. Mucinous pure adenocarcinoma tend to develop a little more frequently at younger ages than mixed version Mm (63.5% in the decades V-VI versus 62.16 in sixth and seventh decades of life).

Regarding gender, the results showed a relatively balanced sex ratio. For the MP variant, the gender ratio was 1.37 in favor of men, while for the MM variant was 1.16 and 1.31 for the Mm. In the case of signet ring variant, the ratio value was slightly higher, 1.6 for males. Compared to the version of rectal adenocarcinoma, the mucinous did not prove to have a developing predisposition towards a certain gender.

Depending on the differentiation degree, moderately and poorly differentiated forms were the most frequent, while the well-differentiated forms were the least encountered.

Considering the histopathological subtypes, we have recorded that moderately differentiated forms were encountered in all mucinous carcinomas types except for the rectal SR form, poorly differentiated forms predominating. Although well-differentiated forms were the most underrepresented, they were more common in the Mm version of mucinous carcinoma.

Regarding the biochemical type of mucin, MP, and SR subtype were characterized by acid mucins and sialomucin, while in the Mm subtype there was a balance of acidic and neutral mucins, namely a slight prevalence of sulfomucin. The prevalence of mucin acids, respectively sulfomucin was characteristic to younger ages. Also an increased ratio of sulfomucin was associated with a decreased differentiation degree and an increased neoplastic stage.

The lymphatic metastasis rate grew as the degree of the tumor differentiation decreased, with a higher disease stage and depth of invasion, being significantly higher in the MP forms compared to the Mm type.

**Fig. 1:**
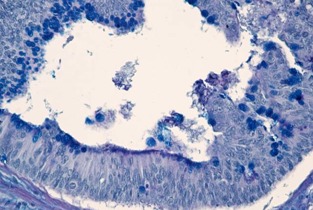
Mucinous rectal adenocarcinoma – Mm. Predominantly acidic intracellular mucin secretion, PAS/ AA stain x 200

**Fig. 2:**
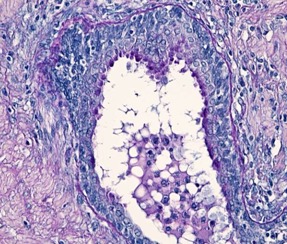
Mucinous rectal adenocarcinoma – Mm. Predominantly neutral mucin secretion PAS/ AA x 200dominantly acidic intracellular mucin secretion, PAS/ AA stain x 200

**Fig. 3:**
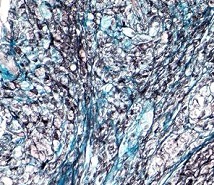
Mucinous rectal adenocarcinoma - Pm. Mixed secretion of sulfomucins and Sialomucins AB/ CI stain x 100

The immunohistochemical profile of mucins showed a higher rate of reactivity of MUC2 and MUC5AC in subtypes MP and SR, while in subtype Mm, MUC1 prevailed. In addition, a decrease in MUC1 reactivity with a higher grade of differentiation and increase of the mucinous component was noticed.

MUC5AC immunostaining was heterogeneous regardless of the histopathological subtype. Such changes in the expression of MUC5AC were recorded at the level of the adenocarcinoma component as well as in the malignant epithelium lining and the mucus lakes. The reaction intensity of this marker was higher mostly in the secretion of the antigen as well as in the mucus lakes and apical cytoplasm of carcinoma goblet cells. We did not find significant correlations between MUC5AC expression and the tumor differentiation degree, pTNM stage and the lymph node metastasis rate. The lack of secretion of MUC5AC was correlated with a poor prognosis for CRC.

**Fig. 4:**
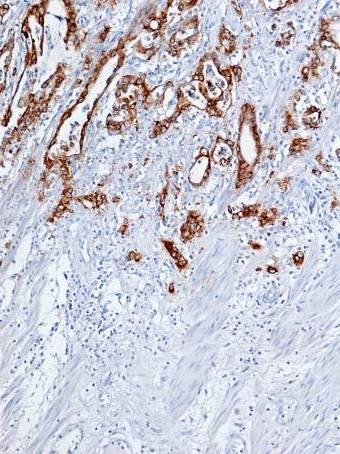
Rectal adenocarcinoma – Mm. Positive cytoplasmic MUC1 stain

**Fig. 5:**
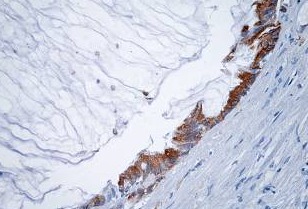
Mucinous rectal adenocarcinoma – PM. Positive MUC2 immunostaining

**Fig. 6:**
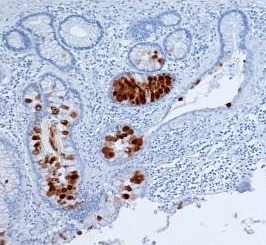
Mucinous rectal adenocarcinoma – Mm. Positive MUC5AC immunostaining in goblet cells cytoplasm

## Discussions

Even though colorectal adenocarcinomas are the most frequent pathological type of tumor encountered within this digestive segment, mucinous carcinoma and signet ring cell carcinomas may occur, with major controversy when discussing their prognosis. With rather different clinical characteristics than adenocarcinoma, while being diagnosed at a higher stage, suggesting a different metastatic pattern and also mostly found in the proximal part of the colon, mucinous adenocarcinoma represents a distinct entity in the CRC tumors. Precise mechanisms of developing MC have not yet been discovered, even though genetic predisposition has been proven to have a higher prevalence in the carcinogenic process [**11**-**14**].

An increased incidence of MC has been encountered in patients with hereditary cancer as well as in inflammatory bowel disease. Moreover, radiotherapy may have unwanted late side effects such as radiation colitis which may develop into CRC [**15**], which confirms the link between radiation induced colitis and the presence of MC [**16**,**17**]. Another particular aspect is that MC seem to be encountered in younger patients [**18**-**20**]. Several studies confirmed that mucinous tumors are more frequent in patients < 39 years old as compared to non-mucinous tumors [**21**]. Our results confirmed that mucinous carcinoma is found in patients with a median age lower than non-mucinous carcinoma, even though most of the patients included in the study were over 50 years old. 

There is no doubt that the pathological examination remains the golden standard and provides a specific and individualized treatment. Characterized by cellular aggregates, which provide a distinctive pattern, diagnosis is not rather difficult; however, what seems to be more significant is that a larger amount of tissue might be necessary to provide a proper diagnosis. Mucinous tumors have a more aggressive behavior, which seems to be caused by their microscopic development [**22**]. Several theories have been taking into account, Sugarbaker’s [**23**] point of view receiving more attention. Apparently, the mucins provide a pressure on the bowel wall and extend further on to the lymphatics. On the other hand, the cellular mucin display may provoke a tumor swelling, due to its ability to imbibe water, pass through the bowel layers, and disseminate from that point on. 

Our study confirmed a different pattern for mucinous rectal cancer that seems to separate it from the non-mucinous types. We highlighted that these tumors are more aggressive, most of them being diagnosed in an advanced stage, possibly due to their location or spreading mechanism. Also the type of secreted mucins may interfere with the prognostic of the disease. Mucin acids and sulfomucin were more frequent in younger ages and the differentiation degree and neoplastic stage were inversely related to the ratio of sulfomucin.

## Conclusions

Rectal carcinomas with mucinous component differ in terms of histological and immunohistochemical profile of mucins. These aspects are useful to establish an effective diagnosis of mucinous carcinoma subtype of localized rectal cancer and to assess the prognostic factors. Even more, this may come handy for a future individualized treatment and provide new scenarios for a better way of early diagnosing CRC. 
